# Práticas integrativas e complementares, hipertensão e indicadores sociais: estudo ecológico, Brasil, 2023

**DOI:** 10.1590/S2237-96222026v35e20250570.pt

**Published:** 2026-10-05

**Authors:** Alexsandra da Silva Ferreira do Nascimento, Maria Carolina Lins de Souza, Stéfane Lele Rossoni, Juliana Henriques dos Santos, Wendell Arthur Lopes, Constanza Pujals, Raíssa Bocchi Pedroso

**Affiliations:** 1 Universidade Estadual de Maringa, Programa de Pós-Graduação em Ciências da Saúde, Maringá, PR, Brasil

**Keywords:** Terapias Complementares, Hipertensão, Fitoterapia, Aromaterapia, Saúde Pública, Terapias Complementarias, Hipertensión, Fitoterapia, Aromaterapia, Salud Pública

## Abstract

**Objetivo:**

Correlacionar o uso de práticas integrativas e complementares (PIC) e os atendimentos de hipertensão, bem como sua correlação com indicadores socioeconômicos no Brasil em 2023.

**Métodos:**

Estudo ecológico com dados do Sistema de Informação em Saúde para a Atenção Básica. Foram analisados os atendimentos de PIC e de hipertensão, além dos indicadores socioeconômicos indice de desenvolvimento humano municipal (IDHM) e produto interno bruto (PIB) per capita. Foram descritas as taxas de PIC e de hipertensão, com aplicação do coeficiente de correlação de Pearson para avaliar as correlações entre as variáveis, além de análise de variância e análise espacial por meio do índice de Moran global e dos indicadores locais de associação espacial.

**Resultados:**

Foram registrados 78.496 atendimentos de PIC, com maior taxa no Rio Grande do Sul (271,75/100 mil habitantes). Para hipertensão, o Ceará registrou a maior taxa (64.409/100 mil habitantes). Observou-se correlação positiva entre PIC e hipertensão (ρ=0,201; p-valor 0,010), bem como com o IDHM (ρ=0,426; p-valor 0,026) e o PIB per capita (ρ=0,443; p-valor 0,020).

**Conclusão:**

Houve correlação positiva entre o uso de PIC, os atendimentos por hipertensão e os indicadores socioeconômicos, o que evidenciou desigualdades regionais na oferta dessas práticas no Brasil.

## Resumen

**Objetivo:**

Correlacionar el uso de prácticas integrativas y complementarias (PIC) y la atención de la hipertensión, así como su correlación con indicadores socioeconómicos en Brasil en 2023.

**Métodos:**

Estudio ecológico con datos del Sistema de Información Sanitaria para la Atención Primaria. Se analizaron las consultas de PIC y de la hipertensión, además de los indicadores socioeconómicos Índice de Desarrollo Humano (IDH) municipal y Producto Interno Bruto (PIB) per cápita. Se describieron las tasas de PIC e hipertensión, aplicando el coeficiente de correlación de Pearson para evaluar las correlaciones entre variables, además de análisis de varianza y análisis espacial mediante el índice global de Moran e indicadores locales de asociación espacial.

**Resultados:**

Se registraron 78.496 consultas de PIC, con la tasa más alta en el estado de Rio Grande do Sul (271,75/100.000 habitantes). Para consultas de hipertensión, el estado de Ceará registró la tasa más alta (64.409/100.000 habitantes). Se observó una correlación positiva entre las PIC y la hipertensión (ρ=0,201; p = 0,010), así como con el IDH (ρ=0,426; p = 0,026) y el PIB per cápita (ρ=0,443; p = 0,020).

**Conclusión:**

Se encontró una correlación positiva entre el uso de PIC, las consultas de hipertensión y los indicadores socioeconómicos, lo que evidencia las desigualdades regionales en la provisión de estas prácticas en Brasil.

## Introdução

A hipertensão é um dos principais desafios globais de saúde pública e afetou 29% dos adultos até 2025, com maior impacto em países em desenvolvimento [1,2]. Considerada a principal causa de morte evitável, a hipertensão responde por 10% a 20% dos óbitos mundiais e representa 10% dos gastos em saúde [3]. No Brasil, sua prevalência varia entre 21% e 35%, com maior incidência em idosos, mulheres e indivíduos com menor escolaridade [4,5]. 

O manejo da hipertensão inclui abordagens não farmacológicas, como mudanças no estilo de vida, telemedicina e monitoramento domiciliar da pressão arterial [6], além de estratégias emergentes, como modulação da microbiota intestinal [7]. 

A adesão ao tratamento farmacológico é limitada pela natureza assintomática da doença [5], o que reforça a necessidade de terapias complementares, como as práticas integrativas complementares. Apesar do potencial delas, ainda há lacunas no entendimento da distribuição e da acessibilidade dessas práticas no contexto da hipertensão, especialmente em relação a disparidades socioeconômicas e regionais. 

As práticas integrativas complementares foram incorporadas ao Sistema Único de Saúde (SUS) em 2006, com ênfase em abordagens holísticas [8]. No Brasil, 6% dos adultos utilizam essas práticas, principalmente mulheres, idosos e indivíduos com maior renda, enquanto populações vulneráveis, como afro-brasileiros e pessoas de baixa renda, apresentaram menor acesso a elas [9,10,11]. 

Estudos demonstraram que práticas integrativas complementares como acupuntura, tai chi chuan e fitoterapia auxiliam no controle da pressão arterial [12], mas a implementação delas tem enfrentado barreiras, como a falta de diretrizes e a variabilidade individual nas respostas [13].

Este estudo objetivou analisar as taxas de atendimentos de práticas integrativas e complementares e os atendimentos individuais para hipertensão no Brasil em 2023, com análise da sua correlação com indicadores socioeconômicos. Buscou-se verificar se os dados de notificações das variáveis investigadas corroboraram os achados da literatura pesquisada sobre o tema.

## Métodos

### Delineamento

Trata-se de estudo ecológico que analisou as taxas de atendimento de práticas integrativas complementares (selecionadas) e a associação com atendimentos individuais de hipertensão. Verificou-se também a correlação dessas variáveis com indicadores socioeconômicos no Brasil em 2023.

### Contexto

A seleção das práticas integrativas e complementares analisadas baseou-se no Mapa de Evidências de Efetividade Clínica das Medicinas Tradicionais, Complementares e Integrativas para Doenças Cardiovasculares. Esse mapa foi desenvolvido através de parceria conjunta entre a Organização Pan-Americana da Saúde, a Organização Mundial da Saúde, o Consórcio Acadêmico Brasileiro de Saúde Integrativa e o Centro Latino-Americano e do Caribe de Informação em Ciências da Saúde. Para sua elaboração, foram incluídas revisões sistemáticas avaliadas pela ferramenta AMSTAR 2, segundo as diretrizes PRISMA e 3iE, com a abrangência de 212 revisões e 37 desfechos clínicos relacionados às doenças cardiovasculares. A partir desse trabalho, selecionamos as práticas integrativas e complementares para este estudo: práticas corporais da medicina tradicional chinesa (tai chi chuan e qi gong) e meditação, que tiveram qualidade alta e efeito potencialmente positivo na hipertensão e na pressão arterial, além da aromaterapia com lavanda e a fitoterapia com Camellia sinensis, com qualidade alta e efeito positivo na pressão arterial [14].

### Participantes

Por se tratar de estudo ecológico, as unidades de análise corresponderam aos estados brasileiros. Foram considerados os registros validados de atendimentos relacionados às práticas integrativas e complementares selecionadas e aos atendimentos individuais para hipertensão disponíveis no Sistema de Informação em Saúde para a Atenção Básica no período de janeiro a dezembro de 2023.

### Variáveis

Foram analisadas as taxas de atendimentos de práticas integrativas e complementares e os atendimentos individuais para hipertensão. Como filtros complementares, para as duas variáveis, foram aplicados os critérios de sexo (masculino e feminino) e de faixa etária, estratificada em três grupos: 18 a 34 anos, 35 a 59 anos e 60 anos ou mais.

Foram utilizados dados de indicadores socioeconômicos extraídos do índice de desenvolvimento humano municipal (IDHM) 2021-2022 [16] e do produto interno bruto (PIB) per capita 2023 [17]. Os dados demográficos foram coletados a partir de bases do Instituto Brasileiro de Geografia e Estatística (IBGE) de 2022-2023 [17].

### Fontes de dados e mensuração

Os dados referentes à utilização das práticas integrativas e complementares em saúde e aos atendimentos individuais para hipertensão foram extraídos do Relatório de Saúde - Produção do Sistema de Informação em Saúde para a Atenção Básica, com a consideração dos registros validados e disponibilizados conforme os critérios operacionais do sistema [15]. A extração foi realizada com unidade geográfica Brasil, agregação por estado e competência de janeiro a dezembro de 2023. Na configuração das variáveis estruturantes do relatório (linha/coluna), utilizou-se a organização por estado como eixo geográfico e por tipo de produção como eixo de categorização dos registros assistenciais, o que permitiu a consolidação das informações por tipo de atendimento e procedimento.

Para a análise das práticas integrativas e complementares, foi selecionado o tipo de produção: procedimento, com aplicação do filtro racionalidade em saúde/procedimentos-práticas integrativas e complementares. Foram incluídos os seguintes procedimentos padronizados: sessão de aromaterapia, tratamento fitoterápico, práticas corporais da medicina tradicional chinesa (tai chi chuan e qi gong) e sessão de meditação.

Para a análise dos atendimentos relacionados à hipertensão, foi selecionado o tipo de produção: atendimento individual, com aplicação do filtro problema/condição avaliada - hipertensão.

### Controle de viés

Para reduzir o viés com relação ao tamanho populacional em cada região, a quantidade de práticas integrativas e complementares realizadas e a quantidade de atendimentos individuais para hipertensão foram ponderadas pela população da faixa etária, sexo e unidade federativa em questão.

### Tamanho do estudo

O estudo incluiu as 27 unidades federativas brasileiras como unidades de análise, considerando os registros disponíveis no Sistema de Informação em Saúde para a Atenção Básica referentes ao período de janeiro a dezembro de 2023.

### Métodos estatísticos

Inicialmente, realizou-se análise descritiva e exploratória das taxas de atendimentos de práticas integrativas e complementares e dos atendimentos individuais para hipertensão nos estados brasileiros.

Em seguida, aplicou-se o coeficiente de correlação de Pearson para analisar as correlações entre as variáveis quantitativas. A análise de variância (ANOVA) foi empregada para avaliar o efeito do sexo, da faixa etária e da interação entre esses fatores sobre as taxas de utilização de práticas integrativas e complementares, considerando os estados brasileiros como unidades de observação. Como teste post-hoc, utilizou-se o teste de Tukey para identificar quais grupos diferem entre si.

Para identificar padrões regionais de similaridade entre os estados brasileiros, conduziram-se duas análises de agrupamento por método hierárquico com distância euclidiana (Figura 1), técnica que permite agrupar unidades com perfis semelhantes e explorar a heterogeneidade espacial dos dados. A primeira análise agrupou os estados segundo o perfil de uso das quatro práticas integrativas e complementares (aromaterapia, tratamentos fitoterápicos, práticas corporais da medicina tradicional chinesa - tai chi chuan e qi gong - e sessões de meditação). A segunda considerou, simultaneamente, as taxas de atendimentos em práticas integrativas e complementares, os atendimentos individuais por hipertensão 

**Figura 1:**
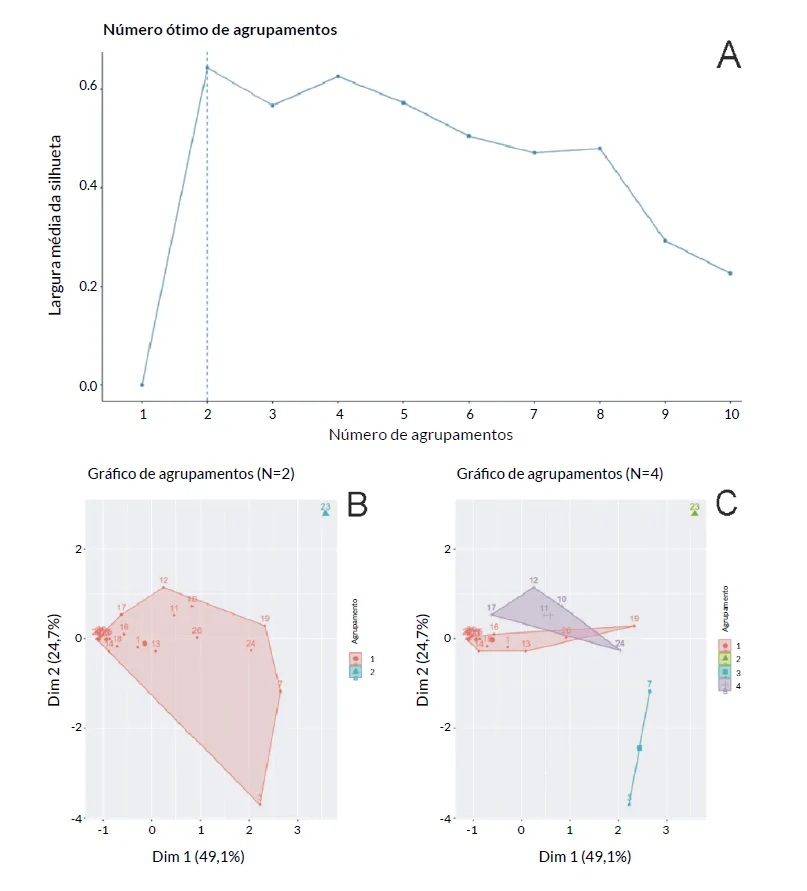

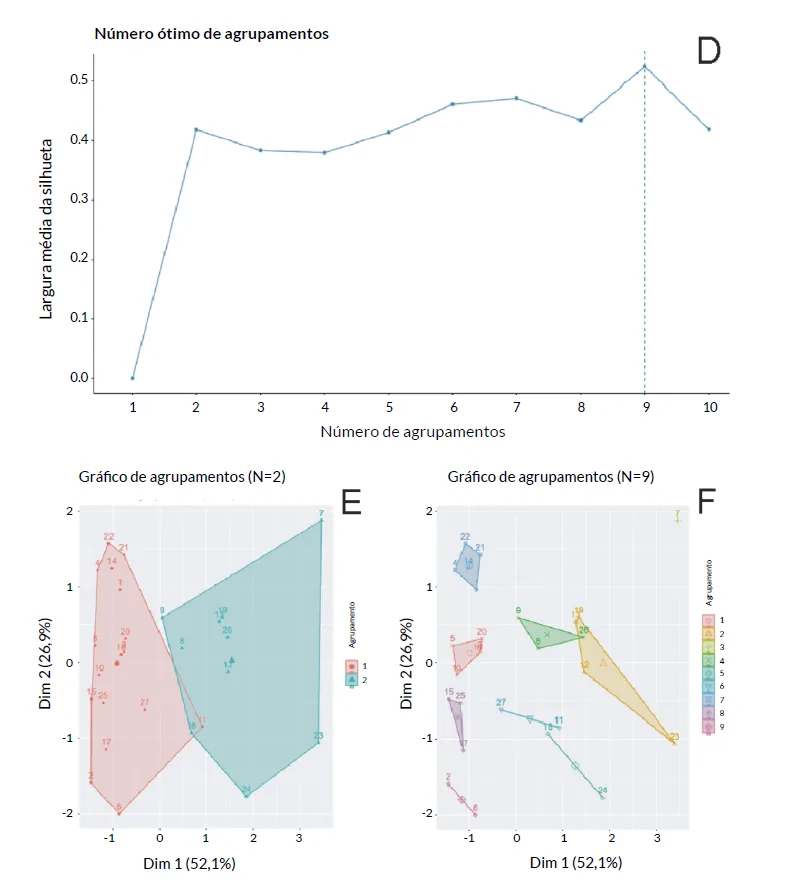
Análise de agrupamento dos estados brasileiros segundo padrões de atendimentos de práticas integrativas e complementares e indicadores associados: número de agrupamentos em função da largura média da silhueta para práticas integrativas e complementares (A); representação gráfica dos agrupamentos com n=2 grupos (B); representação gráfica dos agrupamentos com n=4 grupos (C); número de agrupamentos em função da largura média da silhueta com consideração de práticas integrativas e complementares, hipertensão e indicadores socioeconômicos (D); representação gráfica dos agrupamentos com n=2 grupos (E); representação gráfica dos agrupamentos com n=11 grupos (configuração exploratória) (F)

Legenda: As representações gráficas (B, C, E e F) apresentam a projeção dos estados nas duas primeiras dimensões (dimensão 1 e dimensão 2), que representam os principais eixos de variação dos dados, com sintetização da similaridade entre os estados em relação às variáveis analisadas.

Nota: A análise de agrupamento foi realizada por método hierárquico com distância euclidiana. O número de agrupamentos foi definido com base no maior valor da largura média da silhueta e valores mais elevados indicam melhor separação entre os grupos.

A dimensão espacial foi avaliada por meio do índice de Moran global, de indicadores locais de associação espacial (LISA) e pelo método de estimativa de densidade Kernel. O índice de Moran global foi utilizado para verificar o grau de autocorrelação espacial entre as unidades federativas, o que permitiu identificar se a distribuição das taxas de atendimentos em práticas integrativas e complementares e de hipertensão seguia padrão aleatório, disperso ou agrupado no território. Já os LISA foram empregados para localizar agrupamentos espaciais específicos (alto-alto e baixo-baixo) e anomalias espaciais (alto-baixo e baixo-alto), o que evidenciou áreas onde as taxas de práticas integrativas e complementares ou de hipertensão arterial são semelhantes ou contrastantes em relação às regiões vizinhas. O método de estimativa de densidade Kernel foi aplicado para estimar a densidade espacial dos atendimentos, o que gerou uma superfície contínua que destaca áreas de maior concentração de registros e possíveis hotspots de utilização das práticas integrativas e complementares. Como componente espacial, utilizou-se matriz de contiguidade Queen de ordem 1, e as taxas foram suavizadas com a consideração da média ponderada dos valores dos vizinhos. As análises geoespaciais (Kernel A-B e LISA C-D) estão representadas na Figura 2.

**Figura 2:**
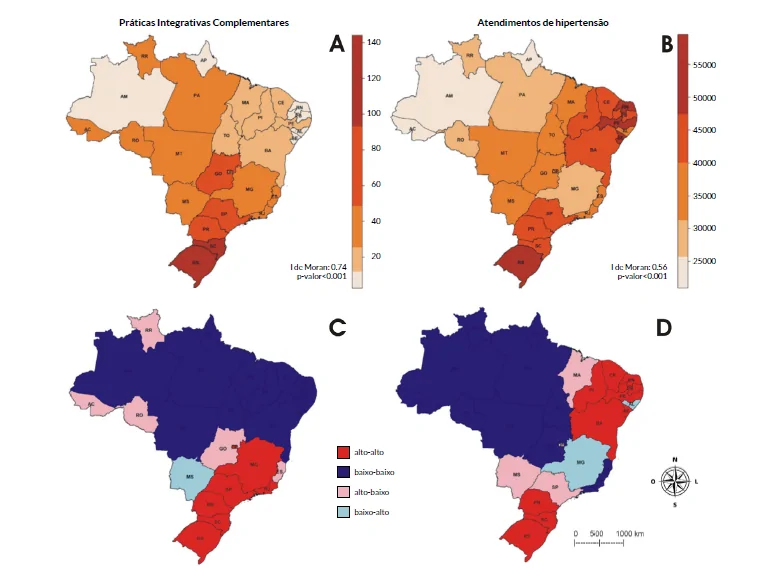
Análise geoespacial da distribuição das práticas integrativas e complementares e dos atendimentos por hipertensão arterial no Brasil: densidade Kernel das taxas de práticas integrativas e complementares (A) e dos atendimentos por hipertensão arterial (B), com evidenciação das áreas de maior concentração dos registros; análise de autocorrelação espacial por meio do índice de Moran global e dos indicadores locais de associação espacial para práticas integrativas e complementares (C) e hipertensão (D), com indicação da presença de aglomerados espaciais

Legenda: Os aglomerados alto-alto representam estados com altas taxas cercados por estados com valores igualmente elevados, enquanto os aglomerados baixo-baixo indicam áreas com baixas taxas circundadas por regiões de baixos valores. Os padrões alto-baixo e baixo-alto indicam discrepâncias espaciais entre um estado e seus vizinhos.

Nota: Apenas os aglomerados estatisticamente significativos (p-valor<0,05) foram considerados na interpretação dos padrões espaciais. A análise foi realizada com utilização da matriz de contiguidade do tipo queen de ordem 1.

Todas as análises estatísticas e geoespaciais foram realizadas no software R [18], com adoção de nível de significância estatística de p-valor<0,05.

## Resultados

Em 2023, no Brasil, foram registradas 78.496 práticas integrativas complementares. Delas, 63,99% eram aromaterapia, 13,89% fitoterapia, 13,39% eram práticas corporais da medicina tradicional chinesa (tai chi chuan e qi gong) e 8,72% correspondiam a meditação. O Rio Grande do Sul destacou-se com a maior taxa de atendimentos, 271,75/100 mil habitantes, seguido pelo Mato Grosso do Sul, com 102,39/100 mil habitantes, e por Santa Catarina, com 93,16/100 mil habitantes. Em contraste, Roraima registrou a menor taxa de atendimento, de 0,44/100 mil habitantes (Tabela 1). 

**Tabela 1:** Classificação estadual das taxas de atendimentos de práticas integrativas e complementares e das taxas de atendimentos por hipertensão por 100 mil habitantes nos estados brasileiros. Brasil, 2023 (n=27)

Unidade Federativa	Taxa de atendimento de práticas integrativas e complementares	Posição das práticas integrativas e complementares	Taxa de atendimento de hipertensão	Posição da hipertensão
Rio Grande do Sul	271,75	1º	40.593,40	10º
Mato Grosso do Sul	102,39	2º	38.064,36	11º
Santa Catarina	93,16	3º	59.553,88	3º
Minas Gerais	76,38	4º	44.954,40	7º
Distrito Federal	74,87	5º	14.675,08	27º
Amazonas	69,52	6º	30.473,24	20º
Rio de Janeiro	56,42	7º	29.698,86	21º
Maranhão	51,00	8º	36.884,58	12º
Piauí	44,25	9º	51.099,65	5º
São Paulo	37,32	10º	31.225,86	19º
Mato Grosso	27,72	11º	36.105,70	13º
Pernambuco	24,81	12º	33.396,58	16º
Paraná	16,24	13º	51.877,98	4º
Acre	13,78	14º	23.016,27	22º
Bahia	7,26	15º	33.834,08	15º
Para	6,33	16º	20.947,52	23º
Rondônia	3,78	17º	18.917,20	25º
Rio Grande do Norte	3,73	18º	32.146,22	17º
Ceará	3,5	19º	64.409,21	1º
Sergipe	3,49	20º	44.907,73	8º
Tocantins	3,10	21º	47.675,20	6º
Alagoas	2,87	22º	60.946,69	2º
Goiás	1,91	23º	31.481,50	18º
Paraíba	1,65	24º	43.494,59	9º
Espírito Santo	1,46	25º	35.919,46	14º
Amapá	1,12	26º	20.566,78	24º
Roraima	0,44	27º	15.532,95	26º

Quanto aos atendimentos para hipertensão, o Ceará registrou a maior taxa, com 64.409/100 mil habitantes, seguido por Alagoas, com 60.946/100 mil habitantes, e por Santa Catarina, com 59.553/100 mil habitantes. O Distrito Federal registrou a menor taxa, com 14.675/100 mil habitantes (Tabela 1).

A análise de agrupamento foi realizada para classificar os estados brasileiros segundo similaridade nas taxas de atendimentos de práticas integrativas complementares, com a consideração das quantidades de realizações de aromaterapia, de tratamentos fitoterápicos, de práticas corporais da medicina tradicional chinesa e de sessões de meditação, ponderadas pela população acima de 18 anos da região, multiplicadas por 100 mil habitantes (Figura 1 A-C). 

A Figura 1A demonstrou n=2 como configuração ótima (silhouette=0,32), porém n=4 como alternativa válida (silhouette=0,28). O agrupamento 1 (Acre, Alagoas, Amapá, Bahia, Ceará, Espírito Santo, Goiás, Mato Grosso, Pará, Paraíba, Pernambuco, Paraná, Rio de Janeiro, Rio Grande do Norte, Rondônia, Roraima, Sergipe, São Paulo e Tocantins) registrou as menores taxas de práticas integrativas complementares. Os agrupamentos 2 (Rio Grande do Sul) e 4 (Maranhão, Minas Gerais, Mato Grosso do Sul, Piauí, Santa Catarina) destacaram-se pelo uso de aromaterapia, seguida por fitoterápicos e por práticas corporais da medicina tradional chinesa. Já o agrupamento 3 (Amazonas e Distrito Federal) mostrou predomínio de práticas corporais da medicina tradicional chinesa, 32/100 mil habitantes, e de fitoterápicos, 27/100 mil habitantes, com uso residual de outras práticas integrativas complementares (Figura 1 C).

Também foi verificada a análise de agrupamento com o intuito de classificar os estados brasileiros por similaridades de atendimentos de práticas integrativas complementares, atendimentos individuais para hipertensão e indicadores sociais como IDHM e PIB per capita (Figura 1 C-F). 

Na Figura 1E (n=2), dois grupos se mostraram revelados claramente: 17 estados (Acre, Alagoas, Amazonas, Amapá, Bahia, Ceará, Maranhão, Minas Gerais, Pará, Paraíba, Pernambuco, Piauí, Rio Grande do Norte, Rondônia, Roraima, Sergipe e Tocantins) com baixa utilização de práticas integrativas complementares (<20/100 mil habitantes), e 10 (Distrito Federal, Espírito Santo, Goiás, Mato Grosso do Sul, Mato Grosso, Paraná, Rio de Janeiro, Rio Grande do Sul, Santa Catarina e São Paulo) com alta utilização (>65/100 mil habitantes). Observou-se maior utilização de práticas integrativas e complementares em estados com melhores indicadores socioeconômicos. Os tamanhos dos marcadores apresentados nas Figuras 1 E-F mostraram serem proporcionais às taxas de atendimentos.

Além dos padrões geográficos identificados na análise de agrupamentos, examinou-se a correlação entre o uso de práticas integrativas complementares e as variáveis socioeconômicas, demográficas e de saúde. As análises evidenciaram correlações positivas e estatisticamente significativas entre os atendimentos de práticas integrativas e complementares e três variáveis: atendimentos individuais por hipertensão (ρ=0,201; p-valor 0,010), índice de desenvolvimento humano municipal (ρ=0,426; p-valor 0,026) e produto interno bruto per capita (ρ=0,443; p-valor 0,020). A análise de variância demonstrou diferenças significativas no uso de práticas integrativas e complementares por sexo (F=15,87; p-valor<0,001), com taxas médias maiores entre mulheres, de 60,69/100 mil habitantes, em comparação aos homens, de 16,92/100 mil habitantes. Não foi observada diferença estatística significativa para a faixa etária (p-valor 0,053). A interação entre idade e sexo também não se mostrou significativa (p-valor 0,624). 

A análise espacial evidenciou padrões de distribuição das práticas integrativas e complementares e dos atendimentos por hipertensão entre os estados brasileiros, conforme apresentado na Figura 2. Os mapas de calor (Figura 2 A-B) revelaram padrões regionais distintos: as maiores taxas de práticas integrativas complementares concentraram-se no Sul (Rio Grande do Sul, Santa Catarina) e no Sudeste (São Paulo, Rio de Janeiro), enquanto o Nordeste registrou as menores adesões. Em contraste, os atendimentos individuais para hipertensão mostraram altas taxas tanto no Sul (Rio Grande do Sul, Santa Catarina e Paraná) quanto no Nordeste (Bahia, Piauí, Ceará, Rio Grande do Norte, Paraíba, Pernambuco e Sergipe). 

A análise de autocorrelação espacial (Figura 2 C-D) evidenciou padrões significativos de agrupamento global para ambas as variáveis (práticas integrativas e complementares: I=0,74; hipertensão: I=0,56; p-valor 0,001). Na análise local, foram considerados apenas os aglomerados estatisticamente significativos (p-valor<0,05), com observação de aglomerados do tipo alto-alto nas regiões Sul/Sudeste para práticas integrativas e complementares e no Sul/Nordeste para hipertensão, e aglomerados baixo-baixo predominantemente no Norte/Nordeste para práticas integrativas e complementares e no Centro-Oeste/Norte para hipertensão. Estados como Rio Grande do Sul, Santa Catarina e Paraná apresentaram altas taxas em ambos os indicadores. Em contraste, o Nordeste registrou baixa utilização de práticas integrativas e complementares associada a elevadas taxas de atendimentos por hipertensão, enquanto o Rio de Janeiro apresentou padrão inverso.

## Discussão

Este estudo identificou que os atendimentos das práticas integrativas complementares no Brasil estão correlacionados a indicadores socioeconômicos (IDHM e PIB per capita) e à demanda por atendimentos individuais para hipertensão, o que serviu para mostrar correlações positivas consistentes. Espacialmente, notou-se maior ocorrência do uso das práticas integrativas complementares em estados ao Sul do Brasil, como Rio Grande do Sul e Santa Catarina, e menor ocorrência entre alguns estados do Nordeste, como Piauí, Ceará, Paraíba, Pernambuco e Alagoas. 

Entre as principais limitações deste estudo, destaca-se o uso exclusivo de dados secundários, o que pode levar a subnotificações e limitações na precisão e na abrangência das informações. Ademais, o estudo focou nos dados da atenção primária à saúde, sem contemplar práticas realizadas em outros níveis de atenção do SUS ou no setor privado. Evidências da literatura indicaram que parte relevante do uso de práticas integrativas ocorre fora da atenção básica, o que pode resultar em subestimação da utilização total dessas práticas no país[22,23]. Também houve ausência de avaliação dos fatores culturais e regionais que influenciam a adoção dessas práticas, o que poderia oferecer uma compreensão mais profunda das desigualdades observadas. Por fim, a utilização de dados agregados limita a generalização dos resultados para grupos específicos da população, como aqueles com diferentes condições socioeconômicas ou de saúde. Essas limitações reforçam a necessidade de estudos complementares, com abordagens longitudinais e qualitativas, para explorar melhor o impacto e os determinantes do uso das práticas integrativas complementares como manejo da hipertensão.

Mesmo com as limitações apontadas, a análise revelou importantes disparidades na adoção das práticas integrativas complementares, com maior prevalência em estados com melhores condições de IDHM e de PIB per capita, sem diferença estatisticamente significativa entre as faixas etárias analisadas. O estudo também trouxe contribuições significativas ao identificar que as práticas integrativas complementares selecionadas estão sendo pesquisadas como ferramentas auxiliares no controle da hipertensão. Esses achados reforçam a relevância das práticas integrativas complementares na promoção da saúde e no manejo de condições crônicas, o que oferece informações úteis para embasar o planejamento de políticas públicas que promovam maior equidade e acessibilidade no uso dessas práticas em todo o país. 

A análise por aglomerados revelou quatro padrões distintos de utilização, com destaque para a predominância da aromaterapia, especialmente no Rio Grande do Sul, que apresentou maiores taxas em relação aos demais estados. A abordagem espacial confirmou concentração no Sul/Sudeste e baixa adesão no Nordeste, com paradoxos relevantes: enquanto estados como Rio Grande do Sul, Santa Catarina e Paraná apresentaram altas taxas de atendimentos tanto de práticas integrativas complementares quanto de hipertensão, o Nordeste mostrou elevadas taxas de hipertensão com baixa utilização de práticas integrativas complementares, e o Rio de Janeiro apresentou o padrão inverso. 

No entanto, de acordo com dados encontrados anteriormente, a região Norte do Brasil teve a maior prevalência de uso de práticas integrativas e complementares, particularmente através do uso de medicamentos fitoterápicos, com prevalência relatada de 3,7% em 2019 [10]. A região Sul também mostrou alta prevalência no uso da medicina tradicional e da medicina complementar, com os residentes mais propensos a se envolverem em práticas como acupuntura e homeopatia. Esse fato se mostrou ligado a maiores níveis educacionais e melhor status socioeconômico na região [10]. Tal informação contradiz com os achados nos dados coletados no atual estudo, onde a prevalência de uso de práticas integrativas complementares registrada para a região Norte no Sistema de Informação em Saúde para a Atenção Básica (SISAB) apresentaram as menores taxas. Embora o uso de medicamentos fitoterápicos tenha sido mais comum na região Norte do Brasil, paradoxalmente, houve disparidade no oferecimento de práticas integrativas complementares na atenção primária à saúde nessa região [10]. Esse fator pode justificar os resultados observados, uma vez que a base de dados utilizada está centrada nesse nível de atenção à saúde.

Em relação às modalidades de práticas integrativas complementares com evidência para hipertensão, a aromaterapia foi a mais empregada, seguida pela fitoterapia, pelas práticas corporais da medicina tradicional chinesa e pela meditação. A análise não identificou diferença estatisticamente significativa entre as faixas etárias (p-valor 0,053), o que indicou padrão semelhante de utilização entre os grupos etários.

Os resultados da Tabela 2 demonstraram correlações positivas entre o uso de práticas integrativas e complementares, os indicadores socioeconômicos e os atendimentos para hipertensão. Esses achados encontraram ressonância na análise espacial, porém com importantes nuances regionais. Enquanto a análise nacional revelou tendências gerais, a abordagem geoespacial evidenciou padrões territoriais específicos: estados com maior desenvolvimento (como Rio Grande do Sul, Santa Catarina e São Paulo) confirmaram-se como aglomerados de alta utilização tanto de atendimentos de práticas integrativas complementares quanto de hipertensão, o que corroborou as correlações positivas identificadas. A análise geoespacial revelou exceções significativas, particularmente no Nordeste, onde elevadas taxas de hipertensão coexistiram com baixa adoção de práticas integrativas e complementares, sugerindo a influência de fatores regionais não capturados pelas correlações nacionais.

**Tabela 2:** Correlação entre atendimentos de práticas integrativas e complementares, indicadores socioeconômicos e variáveis demográficas nos estados brasileiros. Brasil, 2023 (n=27)

Análise	Variáveis comparadas	Estatística de teste	ρ	p-valor
Pearson	Práticas integrativas e complementares × Hipertensão	2,60	0,201	0,010
	Práticas integrativas e complementares × IDHM	2,35	0,426	0,026
	Práticas integrativas e complementares × PIB per capita	2,47	0,443	0,020
ANOVA	Sexo	15,87	-	<0,001
	Faixa etária	2,99	-	0,053
	Sexo: Faixa etária	0,47	-	0,624

Igualmente revelador foi o caso do Rio de Janeiro, que apresentou padrão inverso ao nacional, com alto índice de atendimentos de práticas integrativas complementares e baixos índices de atendimentos por hipertensão. Esses achados destacaram que, embora as correlações nacionais tenham apontado para tendências gerais, a distribuição espacial desses fenômenos foi marcadamente heterogênea, com aglomerados regionais que podem tanto confirmar quanto contradizer os padrões esperados. Tal constatação enfatiza a importância de se considerar tanto as tendências nacionais quanto as particularidades regionais na formulação de políticas públicas em saúde.

Taxas maiores de atendimentos em práticas integrativas e complementares observadas em estados com maior IDHM e PIB per capita podem refletir maior capacidade institucional e fiscal para estruturar a oferta na atenção primária à saúde, com inclusão de contratação e de fixação de profissionais habilitados, de investimento em capacitação e de aquisição de insumos necessários para determinadas modalidades. Evidências recentes indicaram que a implementação dessas práticas no SUS é fortemente influenciada pela priorização na agenda da gestão local e pela disponibilidade de recursos humanos qualificados, o que contribui para padrões territoriais heterogêneos de oferta [19,20].

Já as diferenças regionais podem estar relacionadas à distribuição desigual de oportunidades de formação profissional em saúde integrativa, concentradas em regiões com maior densidade de instituições de ensino e redes assistenciais mais estruturadas, o que tende a ampliar a capacidade local de ofertar e sustentar essas práticas ao longo do tempo [20]. 

Parte das variações observadas também pode decorrer de diferenças na qualidade e no uso dos sistemas de informação em saúde. Estudos recentes apontaram que a completude e a consistência dos registros no SISAB variam entre unidades federativas, especialmente para procedimentos menos consolidados na rotina dos serviços, o que pode resultar em subnotificação e inconsistências regionais nos dados administrativos [21]. 

A discrepância entre uso populacional e atendimentos registrados é igualmente plausível. Análises com dados da Pesquisa Nacional de Saúde indicaram que o uso de práticas integrativas varia segundo características sociodemográficas e regionais, e que parte expressiva desse uso ocorre fora da rede institucional do SUS, por meio de autocuidado, de oferta comunitária e de práticas tradicionais de saúde, sem ser captada pelos sistemas administrativos [22]. Desse modo, observa-se divergência entre a disponibilidade de medicina tradicional e complementar reportada nos registros oficiais da atenção primária à saúde e a oferta efetivamente identificada em estudos específicos. Enquanto dados oficiais indicam que cerca de 20% das equipes de atenção primária à saúde ofertam medicina tradicional e complementar, o estudo realizado por Sousa et al. [23] identificou disponibilidade dessas práticas em apenas 8% dos municípios avaliados. Esse achado sugere possíveis limitações relacionadas aos processos de registro, monitoramento e divulgação dos dados [23].

Os resultados do presente estudo evidenciaram disparidades regionais no acesso às práticas integrativas e complementares, especialmente entre as regiões Sul e Sudeste, que apresentaram maiores taxas de utilização, e as regiões Norte e Nordeste, que registraram menores taxas. Esses achados reforçam a necessidade de políticas públicas que ampliem a oferta dessas práticas em áreas menos assistidas, padronizem os sistemas de registro para aprimorar a vigilância e considerem as particularidades regionais na implementação dessas estratégias. Apesar das limitações inerentes ao uso de dados secundários, este estudo fornece subsídios importantes para orientar a expansão equitativa das práticas integrativas e complementares no SUS, particularmente no contexto do manejo da hipertensão.

## Data Availability

Os bancos de dados utilizados na pesquisa, bem como o código em extensão R, estão disponíveis no link: https://osf.io/8yfhm/overview, na plataforma Open Science Framework.
