# Supplementary material for: Práticas integrativas e complementares, hipertensão e indicadores sociais: estudo ecológico, Brasil, 2023
Source: Epidemiol Serv Saude. 2026 Oct 5;35:e20250570. [Article in Portuguese] doi: 10.1590/S2237-96222026v35e20250570.pt (PMC13647261; doi:10.1590/S2237-96222026v35e20250570.pt)
Supplement: Translation

# Translation: Integrative and complementary practices, hypertension and social indicators: an ecological study, Brazil, 2023

**DOI:** 10.1590/S2237-96222026v35e20250570.en

**Authors:** Alexsandra da Silva Ferreira do Nascimento, Maria Carolina Lins de Souza, Stéfane Lele Rossoni, Juliana Henriques dos Santos, Wendell Arthur Lopes, Constanza Pujals, Raíssa Bocchi Pedroso

**Affiliations:** 1 Universidade Estadual de Maringa, Programa de Pós-Graduação em Ciências da Saúde, Maringá, PR, Brazil

**Keywords:** Complementary Therapies, Hypertension, Phytotherapy, Aromatherapy, Public Health

## Abstract

**Objective:**

To correlate use of integrative and complementary practices (ICP) and hypertension consultations, as well as their correlation with socioeconomic indicators in Brazil in 2023.

**Methods:**

This was an ecological study with data from the Primary Health Care Information System. ICP and hypertension care were analyzed, in addition to the following socioeconomic indicators: municipal human development index (MHDI) and gross domestic product per capita (GDP). ICP and hypertension rates were described, applying Pearson’s correlation coefficient to assess correlations between variables, in addition to analysis of variance and spatial analysis using global Moran’s I and local indicators of spatial association.

**Results:**

78,496 ICP consultations were recorded, with the highest rate in Rio Grande do Sul state (271.75/100,000 inhabitants). Regarding hypertension consultations, Ceará state recorded the highest rate (64,409/100,000 inhabitants). Positive correlation was observed between ICP and hypertension (ρ=0.201; p-value 0.010), as well as with MHDI (ρ=0.426; p-value 0.026) and GDP per capita (ρ=0.443; p-value 0.020).

**Conclusion:**

There was positive correlation between use of PIC, hypertension consultations and socioeconomic indicators, highlighting regional inequalities in the provision of these practices in Brazil.

## Introduction

Hypertension is one of the major global public health challenges and will affect 29% of adults by 2025, with a greater impact in developing countries [1,2]. Considered the leading cause of preventable death, hypertension accounts for 10% to 20% of global deaths and represents 10% of health expenditure [3]. In Brazil, its prevalence varies between 21% and 35%, with higher incidence in the elderly, women and individuals with lower education levels [4,5].

Hypertension management includes non-pharmacological approaches, such as lifestyle changes, telemedicine and home blood pressure monitoring [6], as well as emerging strategies, such as gut microbiota modulation [7].

Adherence to pharmacological treatment is limited by the asymptomatic nature of hypertension [5], which reinforces the need for complementary therapies, such as integrative and complementary practices. Despite their potential, there are still gaps in understanding the distribution and accessibility of these practices in the context of hypertension, especially in relation to socioeconomic and regional disparities.

Integrative and complementary practices were incorporated into the Brazilian Unified Health System (*Sistema* Único *de Saúde*, SUS) in 2006, with an emphasis on holistic approaches [8]. In Brazil, 6% of adults use these practices, mainly women, the elderly and individuals with higher incomes, while vulnerable populations, such as Afro-Brazilians and people with low incomes, have less access to them [9,10,11].

Studies have shown that integrative complementary practices such as acupuncture, tai chi chuan and herbal medicine help control blood pressure [12], but their implementation has faced barriers, such as lack of guidelines and individual variability in responses [13].

This study aimed to analyze the rates of integrative and complementary practice consultations and individual consultations for hypertension in Brazil in 2023, analyzing their correlation with socioeconomic indicators. It sought to verify whether the reporting data for the variables investigated corroborated the findings of the literature reviewed on the subject.

## Methods

### Design

This is an ecological study that analyzed the rates of (selected) integrative and complementary practice consultations and their association with individual hypertension consultations. Correlation between these variables and socioeconomic indicators in Brazil in 2023 was also examined.

### Setting

The selection of the integrative and complementary practices analyzed was based on the Evidence Map of Traditional, Complementary and Integrative Medicine for Cardiovascular Diseases. This map was developed through a joint partnership between the Pan American Health Organization, the World Health Organization, the Brazilian Academic Consortium for Integrative Health, and the Latin American and Caribbean Center on Health Sciences Information. The Map was prepared through inclusion of systematic reviews assessed by the AMSTAR 2 tool, in accordance with the PRISMA and 3iE guidelines, encompassing 212 reviews and 37 clinical outcomes related to cardiovascular diseases. From this work, we selected the integrative and complementary practices for this study: traditional Chinese medicine body practices (tai chi chuan and qi gong) and meditation, which had high quality and a potentially positive effect on hypertension and blood pressure, in addition to aromatherapy with lavender and phytotherapy with Camellia sinensis, with high quality and a positive effect on blood pressure [14].

### Participants

As this is an ecological study, the units of analysis corresponded to the Brazilian states. Validated records of consultations related to the selected integrative and complementary practices and individual hypertension consultations available on the Primary Health Care Information System from January to December 2023 were included in the study.

### Variables

The integrative and complementary practice consultation rates and individual hypertension consultation rates were analyzed. The sex criterion (male and female) and the age group criterion, stratified into three groups, were applied as complementary filters to both variables: 18 to 34 years, 35 to 59 years, and 60 years or more.

Socioeconomic indicator data extracted from the Municipal Human Development Index (MHDI) 2021-2022 data [16] and Gross Domestic Product (GDP) per capita data for the year 2023 [17] were used. Demographic data were collected from the Brazilian Institute of Geography and Statistics (IBGE) databases for 2022-2023 [17].

### Data sources and measurement

Data regarding use of integrative and complementary health practices and individual hypertension consultations were extracted from the Production Report available on the Health Information System for Primary Care, considering validated records made available according to the system’s operational criteria [15]. Data extraction was performed using Brazil as the geographic unit, aggregation by state, and taking the period from January to December 2023. When configuring the report’s structuring variables (row/column), organization by state was used as the geographic axis, while organization by type of production was used as the axis for categorizing care records, which allowed the information to be consolidated by type of consultation and procedure.

In order to analyze integrative and complementary practices, the type of production selected was: procedure, applying the rationality in health/procedures-integrative and complementary practices filter. The following standardized procedures were included: aromatherapy session, phytotherapeutic treatment, traditional Chinese medicine body practices (tai chi chuan and qi gong), and meditation session.

In order to analyze hypertension-related consultations, the selected service type was: individual consultation, applying the filter for the problem/condition being assessed, i.e. hypertension.

### Bias control

In order to reduce bias related to population size in each region of Brazil, the number of integrative and complementary practices performed and the number of individual hypertension consultations were weighted by the population corresponding to the age group, sex, and federative unit/state in question.

### Study size

The study included all 27 Brazilian federative units/states as units of analysis, considering the records available in the Primary Health Care Information System for the period from January to December 2023.

### Statistical methods

Initially, a descriptive and exploratory analysis was conducted of the rates of integrative and complementary practice consultations and individual hypertension consultations in the Brazilian states.

Following this, Pearson’s correlation coefficient was applied to analyze correlations between the quantitative variables. Analysis of variance (ANOVA) was used to assess the effect of sex and age group, and the interaction between these factors on the rates of use of integrative and complementary practices, considering the Brazilian states as units of observation. Tukey’s test was used as a post-hoc test, to identify which groups differed from each other.

In order to identify regional patterns of similarity between Brazilian states, two hierarchical clustering analyses with Euclidean distance were conducted (Figure 1), this being a technique that enables units with similar profiles to be grouped together and also enables the spatial heterogeneity of the data to be explored. The first analysis grouped the states according to the usage profile of the four integrative and complementary practices (aromatherapy, herbal treatments, traditional Chinese medicine body practices - tai chi chuan and qi gong - and meditation sessions). The second considered, simultaneously, integrative and complementary practice consultation rates and individual hypertension consultation rates.

**Figure 1:** Cluster analysis of Brazilian states according to patterns of integrative and complementary practice consultation and associated indicators: number of clusters as a function of average silhouette width for integrative and complementary practices (A); graphical representation of clusters with n=2 groups (B); graphical representation of clusters with n=4 groups (C); number of clusters as a function of average silhouette width considering integrative and complementary practice consultations, hypertension and socioeconomic indicators (D); graphical representation of clusters with n=2 groups (E); graphical representation of clusters with n=11 groups (exploratory configuration) (F)

Legend: Graph representations (B, C, E and F) show the projection of the states in the first two dimensions (dimension 1 and dimension 2), which represent the main axes of data variation, summarizing similarity between the states in relation to the variables analyzed.

Note: Cluster analysis was performed using a hierarchical method with Euclidean distance. The number of clusters was defined based on the largest value of the average silhouette width, with higher values ​​indicating better separation between groups.

The spatial dimension was assessed using global Moran’s I, local indicators of spatial association (LISA), and the Kernel density estimation method. Global Moran’s I was used to verify the degree of spatial autocorrelation between federative units/states, allowing identification of whether the distribution of integrative and complementary practices rates and hypertension rates followed a random, dispersed or clustered pattern in the territory. LISA were used to locate specific spatial clusters (high-high and low-low) and spatial anomalies (high-low and low-high), highlighting areas where the integrative and complementary practices rates or hypertension rates were similar or contrasting in relation to neighboring regions. The Kernel density estimation method was applied to estimate the spatial density of consultations, generating a continuous surface that highlighted areas of higher concentration of records and possible hotspots for the use of integrative and complementary practices. A first-order Queen contiguity matrix was used as a spatial component, and the rates were smoothed by considering the weighted average of the values ​​of neighboring areas. The geospatial analyses (Kernel, A-B and LISA C-D) are represented in Figure 2.

**Figure 2:** Geospatial analysis of the distribution of integrative and complementary practice consultations and hypertension consultations in Brazil: Kernel density of integrative and complementary practice consultation rates (A) and hypertension consultation rates (B), highlighting areas with the highest concentration of records; spatial autocorrelation analysis using the global Moran’s I index and local indicators of spatial association for integrative and complementary practice consultations (C) and hypertension consultations (D), indicating the presence of spatial clusters.

Legend: High-high clusters represent states with high rates surrounded by states with equally high values, while low-low clusters indicate areas with low rates surrounded by regions of low values. High-low and low-high patterns indicate spatial discrepancies between a state and its neighbors.

Note: Only statistically significant clusters (p-value<0.05) were considered in the interpretation of spatial patterns. The analysis was performed using a first-order queen contiguity matrix.

All statistical and geospatial analyses were performed using R software [18], whereby statistical significance was taken to be a p-value<0.05.

## Results

In 2023, 78,496 integrative and complementary practice consultations were registered in Brazil. Of these, 63.99% were aromatherapy, 13.89% phytotherapy, 13.39% were traditional Chinese medicine body practices (tai chi chuan and qi gong), and 8.72% corresponded to meditation. Rio Grande do Sul state stood out with the highest consultion rate, 271.75/100,000 inhabitants, followed by Mato Grosso do Sul state, with 102.39/100,000 inhabitants, and Santa Catarina state, with 93.16/100,000 inhabitants. In contrast, Roraima state registered the lowest consultation rate, namely 0.44/100,000 inhabitants (Table 1).

**Table 1:** State ranking of integrative and complementary practice consultation rates and hypertension consultation rates per 100,000 inhabitants in Brazilian states. Brazil, 2023 (n=27)

Federative Unit/state	Integrative and complementary practice consultation rate	Integrative and complementary practice ranking	Hypertension consultation rate	Hypertension ranking
Rio Grande do Sul	271.75	1^st^	40,593.40	10^th^
Mato Grosso do Sul	102.39	2^nd^	38,064.36	11^th^
Santa Catarina	93.16	3^rd^	59,553.88	3^rd^
Minas Gerais	76.38	4^th^	44,954.40	7^th^
Distrito Federal	74.87	5^th^	14,675.08	27^th^
Amazonas	69.52	6^th^	30,473.24	20^th^
Rio de Janeiro	56.42	7^th^	29,698.86	21^st^
Maranhão	51.00	8^th^	36,884.58	12^th^
Piauí	44.25	9^th^	51,099.65	5^th^
São Paulo	37.32	10^th^	31,225.86	19^th^
Mato Grosso	27.72	11^th^	36,105.70	13^th^
Pernambuco	24.81	12^th^	33,396.58	16^th^
Paraná	16.24	13^th^	51,877.98	4^th^
Acre	13.78	14^th^	23,016.27	22^nd^
Bahia	7.26	15^th^	33,834.08	15^th^
Pará	6.33	16^th^	20,947.52	23th
Rondônia	3.78	17^th^	18,917.20	25^th^
Rio Grande do Norte	3.73	18^th^	32,146.22	17^th^
Ceará	3.5	19^th^	64,409.21	1^st^
Sergipe	3.49	20^th^	44,907.73	8^th^
Tocantins	3.10	21^st^	47,675.20	6^th^
Alagoas	2.87	22^nd^	60,946.69	2^nd^
Goiás	1.91	23^rd^	31,481.50	18^th^
Paraíba	1.65	24^th^	43,494.59	9^th^
Espírito Santo	1.46	25^th^	35,919.46	14^th^
Amapá	1.12	26^th^	20,566.78	24^th^
Roraima	0.44	27^th^	15,532.95	26^th^

Regarding hypertension consultations, Ceará state recorded the highest rate, with 64,409/100,000 inhabitants, followed by Alagoas state, with 60,946/100,000 inhabitants, and Santa Catarina state, with 59,553/100,000 inhabitants. The Federal District recorded the lowest rate, with 14,675/100,000 inhabitants (Table 1).

Cluster analysis was performed to classify Brazilian states according to similarity in the integrative and complementary practice consultation rates, considering the number of aromatherapy sessions, herbal treatments, traditional Chinese medicine body practices, and meditation sessions, weighted by the population over 18 years of age in the region, multiplied by 100,000 inhabitants (Figure 1 A-C).

Figure 1A showed n=2 as the optimal configuration (silhouette=0.32), but n=4 as a valid alternative (silhouette=0.28). Cluster 1 (Acre, Alagoas, Amapá, Bahia, Ceará, Espírito Santo, Goiás, Mato Grosso, Pará, Paraíba, Pernambuco, Paraná, Rio de Janeiro, Rio Grande do Norte, Rondônia, Roraima, Sergipe, São Paulo and Tocantins) recorded the lowest rates of integrative and complementary practices. Clusters 2 (Rio Grande do Sul) and 4 (Maranhão, Minas Gerais, Mato Grosso do Sul, Piauí, Santa Catarina) stood out for the use of aromatherapy, followed by herbal remedies and traditional Chinese medicine body practices. Group 3 (Amazonas and the Federal District) showed a predominance of traditional Chinese medicine body practices, 32/100,000 inhabitants, and herbal remedies, 27/100,000 inhabitants, with residual use of other complementary integrative practices (Figure 1 C).

Cluster analysis was also performed to classify Brazilian states based on similarities in the provision of integrative and complementary practice consultations, individual hypertension consultations, and social indicators such as MHDI and GDP per capita (Figure 1 C-F).

In Figure 1E (n=2), two groups were clearly revealed: 17 states (Acre, Alagoas, Amazonas, Amapá, Bahia, Ceará, Maranhão, Minas Gerais, Pará, Paraíba, Pernambuco, Piauí, Rio Grande do Norte, Rondônia, Roraima Sergipe, and Tocantins) with low use of integrative and complementary practices (<20/100,000 inhabitants), and 10 (Distrito Federal, Espírito Santo, Goiás, Mato Grosso do Sul, Mato Grosso, Paraná, Rio de Janeiro, Rio Grande do Sul, Santa Catarina and São Paulo) with high use (>65/100,000 inhabitants). Higher use of integrative and complementary practices was observed in states with better socioeconomic indicators. The sizes of the markers presented in Figures 1E-F were shown to be proportional to the consultation rates.

In addition to the geographic patterns identified in the cluster analysis, correlation between the use of integrative and complementary practices and socioeconomic, demographic, and health variables was examined. The analyses showed positive and statistically significant correlations between integrative and complementary practice consultations and three variables: individual hypertension consultations (ρ=0.201; p-value 0.010), municipal human development index (ρ=0.426; p-value 0.026), and gross domestic product per capita (ρ=0.443; p-value 0.020). Analysis of variance demonstrated significant differences in the use of integrative and complementary practices by sex (F=15.87; p-value<0.001), with higher average rates among women, at 60.69/100,000 inhabitants, compared to men, at 16.92/100,000 inhabitants. No statistically significant difference was observed for age group (p-value 0.053). Interaction between age and sex was also not significant (p-value 0.624).

The spatial analysis revealed distribution patterns of integrative and complementary practices and hypertension consultations between Brazilian states, as shown in Figure 2. The heat maps (Figure 2 A-B) revealed distinct regional patterns: the highest rates of integrative and complementary practices were concentrated in the South (Rio Grande do Sul, Santa Catarina) and Southeast (São Paulo, Rio de Janeiro), while the Northeast recorded the lowest rates. In contrast, individual hypertension consultations showed high rates in both the South (Rio Grande do Sul, Santa Catarina and Paraná) and the Northeast (Bahia, Piauí, Ceará, Rio Grande do Norte, Paraíba, Pernambuco and Sergipe).

The spatial autocorrelation analysis (Figure 2 C-D) revealed significant global clustering patterns for both variables (integrative and complementary practices: I=0.74; hypertension: I=0.56; p-value 0.001). In the local analysis, only statistically significant clusters (p-value<0.05) were considered, with high-high clusters observed in the South/Southeast regions for integrative and complementary practices and in the South/Northeast for hypertension, and low-low clusters predominantly in the North/Northeast for integrative and complementary practices and in the Midwest/North for hypertension. States such as Rio Grande do Sul, Santa Catarina and Paraná showed high rates for both indicators. In contrast, the Northeast registered low use of integrative and complementary practices associated with high rates of hypertension consultations, while Rio de Janeiro showed the opposite pattern.

## Discussion

This study identified that integrative and complementary practice consultations in Brazil are correlated with socioeconomic indicators (MHDI and GDP per capita) and demand for individual hypertension consultations, which served to show consistent positive correlations. Spatially, higher occurrence of use of integrative and complementary practices was noted in states in southern Brazil, such as Rio Grande do Sul and Santa Catarina, and lower occurrence among some states in the Northeast, such as Piauí, Ceará, Paraíba, Pernambuco and Alagoas.

Standing out among the main limitations of this study is the exclusive use of secondary data, which may lead to underreporting and limitations in the accuracy and scope of the information. Furthermore, the study focused on primary health care data, without considering practices performed at other levels of care within the Brazilian Unified Health System or in the private sector. Evidence from the literature indicates that a considerable part of integrative practice use occurs outside of primary care, which may result in underestimation of the total use of these practices in Brazil [22,23]. Furthermore, there was no assessment of cultural and regional factors that influence adoption of these practices, which could offer a deeper understanding of the inequalities observed. Finally, use of aggregated data limits the generalization of the results to specific population groups, such as those with different socioeconomic or health conditions. These limitations highlight the need for complementary studies, with longitudinal and qualitative approaches, to better explore the impact and determinants of the use of complementary integrative practices in hypertension management.

Despite the limitations noted, the analysis revealed considerable disparities in adoption of integrative and complementary practices, with higher prevalence in states with better MHDI (Health, Quality, and Technology) and GDP per capita, but with no statistically significant differences between the age groups analyzed. The study also made significant contributions by identifying that the selected integrative and complementary practices are being researched as auxiliary tools in the control of hypertension. These findings reinforce the relevance of integrative and complementary practices in health promotion and management of chronic conditions, offering useful information to support the planning of public policies that promote greater equity and accessibility in the use of these practices throughout Brazil.

The cluster analysis revealed four distinct usage patterns, highlighting the predominance of aromatherapy, especially in Rio Grande do Sul, which showed higher rates compared to the other states. The spatial approach confirmed concentration in the South/Southeast and low adherence in the Northeast, with relevant paradoxes: while states like Rio Grande do Sul, Santa Catarina and Paraná showed high consultation rates for both integrative and complementary practices and hypertension, the Northeast showed high hypertension consultation rates with low use of integrative and complementary practices, while Rio de Janeiro presented the opposite pattern.

However, according to previously found data, the Northern region of Brazil had the highest prevalence of integrative and complementary practices, particularly use of herbal medicines, with a reported prevalence rate of 3.7% in 2019 [10]. The Southern region also showed high prevalence of use of traditional and complementary medicine, with its residents more likely to engage in practices such as acupuncture and homeopathy. This fact was linked to higher educational levels and better socioeconomic status in the region [10]. This information contradicts the findings in the data collected in the current study, where the prevalence of integrative and complementary practices recorded for the Northern region on the Primary Health Care Information System showed the lowest rates. Although use of herbal medicines was more common in the Northern region of Brazil, paradoxically, there was a disparity in the provision of integrative and complementary practices in primary health care in this region [10]. This factor may justify the results observed, since the database used is centered on this level of health care.

Regarding the types of integrative and complementary practices with evidence for use in treatment of hypertension, aromatherapy was the most frequently used, followed by phytotherapy, traditional Chinese medicine body practices, and meditation. Our analysis did not identify a statistically significant difference between age groups (p-value 0.053), indicating a similar pattern of use between age groups.

The results shown in Table 2 demonstrate positive correlations between use of integrative and complementary practices, socioeconomic indicators, and hypertension consultations. These findings were reflected in the spatial analysis, although with important regional nuances. While the national analysis revealed general trends, the geospatial approach highlighted specific territorial patterns: more developed states (such as Rio Grande do Sul, Santa Catarina and São Paulo) were confirmed as clusters of high utilization of both integrative and complementary practices and hypertension consultations, corroborating the positive correlations identified. The geospatial analysis revealed significant exceptions, particularly in the Northeast, where high hypertension consultation rates coexisted with low adoption of integrative and complementary practices, suggesting the influence of regional factors not captured by national correlations.

**Table 2:** Correlation between integrative and complementary practice consultations, socioeconomic indicators and demographic variables in the Brazilian states. Brazil, 2023 (n=27)

Analysis	Variables compared	Test statistic	Ρ	p-value
Pearson	Integrative and complementary practices vs. hypertension	2.60	0.201	0.010
	Integrative and complementary practices vs. MHDI	2.35	0.426	0.026
	Integrative and complementary practices vs. GDP per capita	2.47	0.443	0.020
ANOVA	Sex	15.87	-	<0.001
	Age group	2.99	-	0.053
	Sex: Age group	0.47	-	0.624

Equally revealing was the case of Rio de Janeiro, which presented a pattern opposite to the national one, with a high rate of complementary and integrative practices and low hypertension consultation rates. These findings highlighted that, although national correlations pointed to general trends, the spatial distribution of these phenomena was markedly heterogeneous, with regional clusters that can both confirm and contradict expected patterns. This finding emphasizes the importance of considering both national trends and regional particularities when formulating public health policies.

Higher integrative and complementary practice consultation rates observed in states with higher MHDI and GDP per capita may reflect greater institutional and fiscal capacity to structure primary health care provision, including the hiring and retention of qualified health professionals, investment in training, and acquisition of necessary supplies for certain modalities. Recent evidence has indicated that the implementation of these practices in the SUS is strongly influenced by local management agenda prioritization and availability of qualified human resources, which contributes to heterogeneous territorial patterns of provision [19,20]

Regional differences may be related to unequal distribution of opportunities for professional training in integrative health, concentrated in regions with a higher density of educational institutions and more structured care networks, which tends to increase the local capacity to offer and sustain these practices over time [20].

Part of the variations observed may also stem from differences in health information system quality and use. Recent studies have shown that the completeness and consistency of records held on the Primary Health Care Information System vary between federative units/states, especially for procedures that are less consolidated in service routines, which may result in underreporting and regional inconsistencies in administrative data [21].

The discrepancy between population use and recorded consultations is equally plausible. Analyses of National Health Survey data indicated that use of integrative practices varies according to sociodemographic and regional characteristics, and that a considerable part of their use occurs outside the SUS institutional network, through self-care, community provision and traditional health practices, without being captured by administrative systems [22]. Thus, a divergence is observed between availability of traditional and complementary medicine reported on official primary health care records and its provision actually identified in specific studies. While official data indicate that approximately 20% of primary health care teams offer traditional and complementary medicine, the study conducted by Sousa et al. [23] identified that these practices were available in only 8% of the municipalities they assessed. This finding suggests possible limitations related to data recording, monitoring and dissemination processes [23].

The results of this study revealed regional disparities in access to integrative and complementary practices, especially between the South and Southeast regions of Brazil, which showed higher rates of use, and the North and Northeast regions, which recorded lower rates. These findings emphasize the need for public policies that expand provision of these practices in underserved areas, the need to standardize recording systems to improve surveillance, and the need to consider regional particularities in the implementation of these strategies. Despite the limitations inherent to use of secondary data, this study provides important support for guiding the equitable expansion of integrative and complementary practices in the Brazilian Unified Health System, particularly in the context of hypertension management.

## Data Availability

The databases used in the research, as well as the R code extension, are available at the following link: https://osf.io/8yfhm/overview, on the Open Science Framework platform.
